# Effects of breast milk on Behçet’s disease clinical features

**DOI:** 10.55730/1300-0144.5565

**Published:** 2023-01-04

**Authors:** Hatice Ecem KONAK, Abdulsamet ERDEN, Berkan ARMAĞAN, Serdar Can GÜVEN, Hakan APAYDIN, Pınar Akyüz DAĞLI, Yağnur UZUN, Merve KAYGISIZ, Orhan KÜÇÜKŞAHİN, Ahmet OMMA

**Affiliations:** 1 Clinic of Rheumatology, Ministry of Health Ankara City Hospital, Ankara, Turkey; 2 Department of Internal Medicine, Division of Rheumatology, Faculty of Medicine, Ankara Yıldırım Beyazıt University, Ankara, Turkey; 3 Department of Internal Medicine, Ministry of Health Ankara City Hospital, Ankara, Turkey; 4 Division of Rheumatology, Department of Internal Medicine, Faculty of Medicine, Health Sciences University, Ankara City Hospital, Ankara, Turkey

**Keywords:** Behçet’s disease, breastfeeding, innate immunity, sacroiliitis

## Abstract

**Background/aim:**

The etiology of Behçet’s disease (BD) is not clearly known, however, abnormal activity in T helper (Th) 1, Th 17, and regulatory T cells (Treg) has critical importance in pathogenesis. It has been shown that the intestinal microbiome can be effective in the modulation of these immune abnormalities in BD patients. Breastfeeding increases the maturation of the infant’s intestinal permeability by affecting the newborn’s immature intestinal microbiome and metagenome. We aimed to examine the effects of breastfeeding on disease related symptoms, organ involvements and course of the disease in BD patients.

**Materials and methods:**

This study was designed as a cross-sectional study in Ankara City Hospital rheumatology clinic between December 2021 and March 2022. Patients who were diagnosed with BD by meeting the criteria of the ‘International Study Group’ and whose information we could access by agreeing to participate in the study were enrolled. The mothers of the patients were also contacted and asked whether these patients were breastfed, the duration of breastfeeding, and the mode of birth. Demographic and clinical data of the patients, comorbid diseases, and drugs used for BD were collected from the records in the hospital database. The presence of sacroiliitis in patients was evaluated with sacroiliac X-ray and/or magnetic resonance imaging (MRI), which was requested because of low back pain symptoms and only patients with previous sacroiliac imaging for low back pain were included in the study. BD-related organ damage was measured by the Vasculitis Damage Index (VDI) and Behçet’s syndrome Overall Damage Index (BODI) scores.

**Results:**

A total of 304 patients were included in the study. The percentage of patients who were reported to have ever breastfed (median duration (IQR): 12(12) months, 33.5% < 6 months, 66.4% ≥ 6 months, and 59.6% ≥ 12 months) is 92%. When the breastfed and nonbreastfed patients were compared, 6.8% of the breastfed patients needed TNF-i against 18.2% of the nonbreastfed patients (p = 0.052). While the rate of having at least one comorbidity was 26.4% for those who were breastfed, this rate was 50% for those who had never been breastfed. When the organ and system involvements of the patients were compared, the incidence of sacroiliitis was statistically significantly higher in the nonbreastfed group (p = 0.025). Patients who were breastfed for less than 6 months were diagnosed with BD at an earlier age than those who were breastfed for more than 6 months, and those who were breastfed for less than 12 months compared to those who were breastfed for more than 12 months (respectively, p = 0.039, p = 0.035).

**Conclusion:**

Our results imply that history of breastfeeding may have some positive effects on the course of the disease in BD patients.

## 1. Introduction

Behçet’s disease (BD) is a chronic vasculitic disordercharacterized by recurrent oral and genital ulcers with orwithout the involvement of various other organ systems[1]. The etiology of the disease is not fully clarified,however dysregulated autoimmunity associated withgenetic and environmental factors is thought to play a rolein the pathogenesis [2]. Abnormal activity in T helper (Th) 1, Th 17, and regulatory T cells (Treg) is critical regardingthe autoimmune dysregulation in BD [3,4]. Several studies have reported that the intestinal microbiome, which is an important component of the immune system, can be effective in the modulation of Th1, Th17, and Treg cells and related immune alterations in BD [5–7].

Breast milk is a major contributor to the innate immune defense of the newborn. The newborn’s immune system is immature at birth and many of the innate components of mucosal immunity are not fully developed [8]. Breast milk enhances the intestinal mucosal barrier, increasing the maturation of the infant’s intestinal permeability by regulating immature gut microbiome and metagenome [9–11]. In particular, the bacterial composition of breast milk prevents the emergence of metabolic and autoimmune diseases by ameliorating the host response and potentially preventing abnormal colonization with long-term effects [12]. In many studies, it has been shown that breastfeeding can have protective effects on chronic diseases such as type-2 diabetes mellitus (DM), hypertension (HT), hyperlipidemia (HL), and autoimmune diseases such as inflammatory bowel disease, juvenile idiopathic arthritis, rheumatoid arthritis, and ankylosing spondylitis (AS) [13–20]. Although there is limited data regarding the protective effect of breastfeeding on several chronic rheumatic diseases, to our best knowledge, the relationship between BD and breastfeeding has not been studied yet. In this study, we aimed to examine the effects of breastfeeding on disease-related symptoms, organ involvements and disease course in patients with BD.

## 2. Material and methods

### 2.1. Patients

This study was designed as a cross-sectional study and conducted in Ankara Bilkent City Hospital rheumatology clinic between December 2021 and March 2022. An official permission was obtained from the institutional ethics committee (E2-21-1008), to conduct this study dated 10 November 2021. The study was conducted in accordance with the principles of the Declaration of Helsinki. Adult BD patients followed in our clinic were evaluated using hospital records and a retrospective cohort of 450 patients who met the criteria of the ‘International Study Group’ was formed [21].

### 2.2. Study design

Patients were reached via telephone and surveyed upon verbal consent to participate. The parents of the patients were also contacted and questioned whether these patients received breast milk in their infancy and for how long, in addition to the mode of delivery (vaginal delivery/cesarean section). Patients who did not agree to participate and those whose breast milk intake history could not be recalled were excluded from the study. Patients who received breast milk for more than 48 h were accepted as breastfed [16]. Demographic and clinical data of the patients were collected from the hospital records. Comorbid diseases, BD involvements and treatments, human leukocyte antigen (HLA) B51, and pathergy skin test results were recorded. The presence of sacroiliitis in patients was evaluated with sacroiliac X-ray and/or magnetic resonance imaging (MRI), which was requested because of low back pain symptoms. Patients without a previous sacroiliac joint imaging have been excluded from the study. BD related damage was quantified with the vasculitis damage index (VDI) and Behçet’s Syndrome Overall Damage Index (BODI), an index specifically designed for BD. VDI is a widely used index to evaluate damage in vasculitides. It consists of 64 items based on the scoring system obtained from the individual items from the onset of the disease (grouped into 11 organ-based systems). All items contribute equally to the score. In the index, organ damage is defined as organ pathology that lasts longer than 3 months, especially after the onset of vasculitis symptoms [23]. The VDI scores of the patients included in our study were calculated and those with VDI ≥ 1 were recorded as patients with basal organ damage. BODI is a scale specifically designed to identify and measure organ damage in BD patients. The BODI consists of 34 items categorized into 9 organ/system areas: mucocutaneous, musculoskeletal, ocular, vascular, cardiovascular, neuropsychiatric, gastrointestinal, reproductive, and other. Each item and subitem receive 1 point and the total score ranges from 0 to 46. Organ damage was defined as pathologies lasting longer than 6 months [23]. In our study, BD patients with BODI ≥ 1 were recorded as patients with basal organ damage.

### 2.3. Statistical analysis

Statistical Package for Social Sciences (SPSS) v21 software was used for all statistical evaluations (IBM Corp., Armonk, NY). The conformity of the data to the normal distribution was checked with the Shapiro-Wilk test. The distribution of quantitative data was expressed with means ± standard deviations (SD). Variables that did not fit the normal distribution were expressed with median and interquartile range (IQR) values. The quantitative data was compared between groups using the Student’s t test in case of normal distribution, otherwise using the Mann-Whitney U test. Categorical variables were presented with numbers and percentages and compared using the Chi-Square test.

The effects of different variables on the presence of breastfeeding history in BD patients were evaluated by univariate analysis and the variables for which the unadjusted p-value was <0.10 in logistic regression analysis were identified as potential risk markers and included in the full model. We reduced the model by using backward conditional elimination multivariate logistic regression analyses and eliminated potential risk markers by using likelihood ratio tests. Odds ratio (OR) and relative risk (RR) values and their 95% CI were calculated through crosstabs. p values below 0.05 were considered statistically significant in all tests.

## 3. Results

A total of 450 patients were evaluated for eligibility. One hundred and forty-six patients were excluded due to incomplete medical records or refusal to participate. A total of 304 BD patients were included in the study. Mean ± SD age of the subjects was 40.8 ± 11.1 years, and 46% were female. Two hundred and eighty (92%) patients were breastfed and median (IQR) breastfeeding time was 12 (12) months. Among these, 33.5% were breastfed for < 6 months, 66.4% for ≥ 6 months, and 59.6% for ≥ 12 months. Demographic and clinical data of the patients were presented in Table 1.

When breastfed and nonbreastfed groups were compared, there were no significant differences in terms of age, gender, symptom and diagnosis age, disease duration, disease manifestations, rate of pathergy and HLA-B51 positivity, delivery type, VDI and BODI scores (Table 1). When BD treatments were compared, 6.8% of the breastfed group ever administered tumor necrosis factor alpha (TNFa) inhibitors in comparison to 18.2% of the nonbreastfed group (p = 0.052). The rate of having at least one comorbidity in breastfed patients was 26.4%, whereas it was 50% in the nonbreastfed group (p = 0.014). Among these comorbidities, the incidence of chronic obstructive pulmonary disease (COPD) and asthma was significantly higher in the nonbreastfed group (p = 0.002). When the organ and system involvements due to BD were compared, the incidence of sacroiliitis was significantly higher in the nonbreastfed group (p = 0.025). There was no significant difference between the two groups in terms of other organ/system involvements.

The patients were divided into two groups as <6 months and ≥6 months according to the duration of breastfeeding. There was no significant difference between the two groups in terms of age, gender, symptom age, disease duration, BD-related organ involvement, presence of at least one comorbidity, rates of pathergy and HLA-B51 positivity, mode of delivery, VDI and BODI scores (Table 2). It was observed that the group that was breastfed for <6 months was diagnosed with BD earlier (age of diagnosis, years, mean ± SD: 28.2 ± 8.93 vs. 30.6 ± 9.6, p = 0.039). Likewise, when the patients were divided into two groups as <12 months and ≥12 months according to the duration of breastfeeding, age of diagnosis was younger in the group breastfed <12 months (age of diagnosis, years, mean ± SD: 28.39 ± 9.05 vs. 30.77 ± 9.64, p = 0.035) (Table 2). Furthermore, the incidence of COPD/asthma was significantly higher in the group breastfed for <12 months (p = 0.03) (Table 2).

The association of different variables with a history of breastfeeding in BD was assessed in univariate and multivariate analyses. In univariate analyses, OR for breastfeeding history with TNFa inhibitor usage was 0.364 (95% CI: 0.113–1.173, p = 0.091), OR with sacroiliitis was 0.308 (95% CI: 0.105–0.908, p = 0.033) and OR with comorbidity was 0.359 (95% CI: 0.155–0.835, p = 0.017) (Table 3).

Multivariate logistic regression analysis for the presence of breastfeeding comprising the variables of sacroiliitis, comorbidity, and TNFa inhibitor usage was performed thereafter. In the multivariate analyses, sacroiliitis in the highest tertile (OR: 0.276, 95% CI: 0.091–0.836, p = 0.023) was determined as an independent predictor of breastfeeding history in BD, as well as the presence of comorbidity (OR 0.337, <95% confidence interval 0.143–0.794; p = 0.013) (Table 3). Sacroiliitis occurred in 21 of 280 patients (7.5%) in the breastfed group and in 5 of 24 patients (20.8%) in the nonbreastfed group with a significant difference between groups (RR with 95% CI = 0.36 [0.14–0.86], p = 0.025). Comorbidity occurred in 74 of 280 patients (26.4%) in the breastfed group and in 12 of 24 patients (50%) in the nonbreastfed group with a significant difference between groups (RR with 95% CI = 0.52 [0.33–0.82], p = 0.014).

The relative risk reduction for sacroiliitis in the breastfed group when compared to nonbreastfed group was 64% and the number needed to treat to avert sacroiliitis was 8 (95% CI, 3 to 85). The relative risk reduction for comorbidity with the breastfed group when compared to nonbreastfed group was 47.1% and the number needed to treat to avert comorbidity was 4 (95% CI, 2 to 24).

## 4. Discussion

Our results demonstrated that the presence of at least one comorbidity was less in breastfed BD patients than in nonbreastfed BD patients. In addition, the frequency of COPD/asthma was found to be less in breastfed BD patients. The incidence of sacroiliitis decreased with breastfeeding, regardless of the duration of breastfeeding. Shorter breastfeeding duration seemed to be related to an earlier diagnosis of BD. The need for TNFa inhibitor treatment was more common in nonbreastfed group. The relative risk reduction for sacroiliitis and comorbidity with breastfeeding were 64% and 47.1%, respectively. There has been no previous study regarding the relationship between breast milk and BD, so this study is the first of its kind, to our best knowledge.

Breastfeeding has both protective effects during infancy and in the long-term. Breast milk contains many bioactive components, including immunoglobulins, fatty acids, hormones, and cytokines. Studies have shown that it is both prebiotic and probiotic because it contains oligosaccharides and some bacteria [24]. Breast milk can regulate infant gut microbiota composition indirectly by promoting the growth of certain bacteria through the transfer of prebiotics, but also directly through vertical transmission of bacteria [25,26]. The gut microbiota established in early life affects the health of the individual and also in adulthood. It has been demonstrated that breastfeeding can play a protective role against obesity, HT, HL, and type 2 DM [13,14]. In a study, it was proven that the risk of obesity decreases by 4% with each extra month of breastfeeding [27]. Regarding HT, a prospective study that evaluated preterm infants and followed them through adolescence found that breastfed infants had significantly lower mean diastolic blood pressures than formula-fed infants [28]. A meta-analysis concluded that adults who were breastfed in infancy had a reduction in diastolic blood pressure greater than 0.5 mm Hg compared to formula-fed adults. In addition, adults who were breastfed as infants had a 7 mg/dL reduction in total cholesterol and 7.7 mg/dL in low-density lipoprotein (LDL) cholesterol compared to those who were not breastfed [29]. In addition, it was observed that the risk of developing DM in the first 20 years of life was 33% higher in nonbreastfed infants [30]. In our study, it was found that the relative risk reduction for comorbidity with the breastfed group when compared to the nonbreastfed group was 47.14%, implying that breastfeeding may have protected BD patients from any comorbidity. However, this protective effect was observed independent of the breastfeeding duration.

Breastfeeding seems to be associated with lung health through many mechanisms, including modulation of the gut microbiota, epigenetics, immunity, and lung development. In particular, the bioactive components in breast milk enhance lung health and reduce the risk of asthma by optimizing infant nutrition [31]. It has been shown in many studies that breastfeeding reduces the risk of asthma and allergy, especially in childhood [20]. In a cross-sectional study, it was concluded that exclusive breastfeeding for 3–6 months and partial or exclusive breastfeeding for more than 6 months significantly reduced the risk of asthma [32]. Similarly, in our study, we found that the frequency of COPD/asthma was significantly higher in nonbreastfed BD patients.

Bone marrow edema of the sacroiliac joints is actually considered to be a distinctive feature for spondyloarthritis (SpA) spectrum disorders, however, sacroiliac joint involvement can also be seen in BD [33]. Studies have reported that the frequency of sacroiliitis in Behçet’s disease is between 0%–10% [34]. Since BD mostly affects young male patients similar to SpA, some suggest sacroiliitis in BD may arise from the coexistence of these two conditions [35]. In our study, the incidence of sacroiliitis was found to be 8.55%. This rate was similar to the results in the literature. However, it should be kept in mind that this rate cannot reflect the true prevalence of sacroiliitis associated with BD, since sacroiliac imaging was performed only in patients with symptoms in our study. Van Praet et al. revealed the relation between sacroiliitis, axial involvement, disease activity, and chronic gut inflammation, suggesting a link between gut mucosa inflammation and axial SpA [36]. Breastfeeding in infancy may hypothetically be protective of axial disease via long-term regulatory effects on gut health. Accordingly, in a study evaluating the relationship between breastfeeding and AS development, rate of breastfeeding in infancy in AS patients was found to be lower than their healthy siblings and unrelated healthy controls [16]. It was argued that the effects of breastfeeding on AS development may comprise the interaction of breast milk proteins or lipids with the infant’s immune system directly or indirectly due to their effect on the infant’s intestinal flora dependent immune system. On the other hand, proteins found in formulas given to nonbreastfed infants or other nonbacterial factors may contribute to the development of AS. In our study, sacroiliitis was observed at a rate of 7.5% in breastfed BD patients, while this rate was 20.8% in those who were never breastfed and the relative risk reduction for sacroiliitis with breastfeeding was 64%. However, this frequency of sacroiliitis was independent of the breastfeeding duration. This suggests that breastfeeding, even for a period shorter than 6 months, may be effective in protecting BD patients from sacroiliac joint involvement.

BD patients with life and organ-threatening manifestations or with disease refractory to conventional treatment agents, substantially benefit from TNFa inhibitors [37]. TNFa inhibitor administration was more frequent in nonbreastfed patients in our study, although the difference was not statistically significant. Yet, this result may be speculated to be representative of a more severe and/or refractory disease course in the nonbreastfed group.

The differentiation of T helper cells and corresponding expression of inflammatory cytokines are abnormal in patients with BD. Several studies demonstrated that the number of T regulatory cells and expression of major antiinflammatory cytokines interleukin (IL) 10 and tissue growth factor (TGF) β are decreased in patients with BD. In addition, the ratio of Th17/Treg cells and IL-17 and IL-23 levels significantly increased in these patients, which is thought to be associated with disease activation [38–41]. Studies have confirmed that changes in intestinal microorganisms contribute to the formation and development of BD by influencing Tregs, and that external environmental factors also affect the expression of Th17/Treg ratio which is closely related to BD [40]. Breastfeeding during infancy the Th17/Tregs ratio by ensuring the maturation of the intestinal mucosa. The reason why the breastfed BD patients in our study were diagnosed at a later age can be explained by this regulatory effect of breastfeeding on the immune system.

There are some limitations of the study to be mentioned. Firstly, the study included a relatively small number of BD patients, therefore, the study may be underpowered to obtain more significant results. Secondly, the effects of several confounding factors on our results such as treatment agents, genetic and environmental factors, could not be ruled out. The other limitation of the study is the antibiotics and nutritional factors used during infancy also affect the intestinal microbiome. In addition, the recall bias of the patients in our study is an important problem for the history of breastfeeding. Finally, although the prevalence of COPD/asthma is high in nonbreastfed BD patients, knowledge regarding smoking status of the subjects, which is a major risk factor for obstructive lung disease, is absent.

In conclusion, our results imply that breastfeeding during infancy may have some beneficial effects on the course of the disease in BD. However, there is a need for multicenter studies with a larger number of patients with better elimination of possible confounders to further elucidate the relationship between breastfeeding and BD.

## Figures and Tables

**Table 1:** Demographic and clinical characteristics of patients and comparison according to breastfeeding.

	All patients, n: 304	Breastfed group, n = 280	Non-Breastfed group, n = 24	p
**Female, n (%)**	140 (46)	128 (46)	12 (50)	0.686
**Age, years, mean (SD)**	40.8 (11.1)	40.7 (11.1)	42.1 (10.8)	0.558
**Age of diagnosis, years, mean (SD)**	26.7 (9.9)	29.8 (9.4)	29.9 (8.1)	0.970
**Age of symptom onset, years, median (IQR)**	28.5(13)	25 (13.8)	25.5(11.8)	0.670
**Disease duration, months, median (IQR)**	120 (139)	120 (139)	126 (153)	0.483
**Comorbidity, n (%)**	86 (28.2)	74 (26.4)	12 (50)	**0.014**
**Diabetes mellitu**s	23 (7.5)	20 (7.1)	3 (12.5)	0.341
**Hypertension**	56 (18)	50(18)	6 (25)	0.386
**Coronary artery disease**	21 (6.9)	19 (6.8)	2 (8.3)	0.774
**Chronic kidney disease**	7 (2.3)	5 (1.8)	2 (8.3)	0.090
**COPD-Asthma**	25 (8.2)	19 (6.8)	6 (25.0)	0.002
**Organ involvement due to BD**	184 (60.5)		13 (54.2)	0.507
**Organ and system involvements, n (%)**				
**Oral aphthae**	299 (98.3)	275 (98.2)	24 (100)	1
**Genital aphthae**	241 (79.2)	221 (78.9)	20 (83.3)	0.609
**Papulopustular eruptıon**	229 (75.3)	210 (75.0)	19 (79.2)	0.650
**Erythema nodosum**	139 (45.7)	131 (46.8)	8 (33.3)	0.204
**Arthralgia**	227 (74.6)	209 (74.6)	18 (75.0)	0.960
**Uveitis**	132 (43.4)	120 (42.9)	12 (50.0)	0,498
**Neurological involvement**	21 (6.9)	19 (6.8)	2 (8.3)	0.774
**GIS involvement**	14 (4.65)	13 (4.6)	1 (4.2)	1
**Cardiac involvement**	6 (1.97)	6 (2.1)	0	1
**Thrombosis**	91 (29.9)	83 (29.6)	8 (33.3)	0.705
***Arterial thrombosis***	15 (4.9)	13 (4.6)	2 (8.3)	0.423
***Venous thrombosis***	72 (23.6)	68 (24.3)	4 (16.7)	0.616
**Aneurysm**	14 (4.6)	14 (5)	0	0.612
**Arthritis**	101 (33.2)	93 (33.2)	8 (33.3)	0.990
**Sacroiliitis**	26 (8.55)	21 (7.5)	5 (20.8)	**0.025**
**Articular involvement** *	114 (37.5)	104 (37.1)	10 (41.7)	0.66
**Pathergy positivity, n (%)** **	123 (40.4)	105 (42.3)	9 (39.1)	0.766
**HLA-B51 positivity, n (%)** ***	48 (15.7)	44 (24)	2 (14.3)	0.406
**Family history for BD, n (%)**	82 (26.9)	75 (26.8)	7 (29.2)	0.812
**Medical treatments for BD, n (%)**				
**Colchicine**	229 (75.3)	214 (76.4)	15 (62.5)	0.129
**Azathioprine**	93 (30.5)	89 (31.8)	4 (16.7)	0.123
**Cyclosporine**	6 (1.97)	6 (2.1)	0 (0)	1
**TNFa inhibitor**	25 (8.2)	19 (6.8)	4 (18.2)	0.052
**Vaginal delivery, n (%)** ****	284 (93.4)	264 (94.3)	20 (90.9)	0.519
**VDI score, median (IQR)**	0 (1)	0 (1)	0 (0.75)	0.123
**BODI score, median (IQR)**	0 (1)	0 (1)	0 (0.75)	0.095

* Articular involvement: arthritis and sacroiliitis,

** Pathergy test of 271 patients is known,

*** HLA-B51 test of 197 patients is known,

**** Type of delivery of 302 patients is known,

n: number, min–max: minimum–maximum, SD: standard deviation, IQR: interquartile range, BD: Behçet’s disease, HLAB51: human leukocyte antigen B51, COPD; chronic obstructive pulmonary disease, GIS: gastrointestinal system, TNFa: tumor necrosis factor alpha, VDI: Vasculitis damage index, BODI: Behçet’s Syndrome Overall Damage Index

**Table 2:** Comparison of the Behçet disease patients according to breastfeeding duration.

	Group 1, n=292			Group 2, n=292		
	Breastfed < 6 months, n = 98	Breastfed ≥ 6 months, n = 194	p	Breastfed < 12 months, n = 118	Breastfed ≥ 12 months, n = 174	p
**Female, n (%)**	46(46.9)	91(46.9)	0.99	57(48.3)	80(46)	0.696
**Age of diagnosis, years, mean (±SD)**	28.2(8.93)	30.6(9.6)	0.039	28.39(9.05)	30.77(9.64)	0.035
**Age of symptom onset, years, median (IQR)**	25(10)	25(16)	0.596	24(12.3)	25(14.5)	0.385
**Disease duration, months, median (IQR)**	102(110)	120(144)	0.308	96(108)	120(144)	0.08
**Comorbidity, n(%)**	29(29.6)	55(28.4)	0.825	34(28.8)	50(28.7)	0.988
**Diabetes mellitus**	5(5.1)	18(9.3)	0.211	7(5.9)	16(9.2)	0.310
**Hypertension**	17(17.3)	39(20.1)	0.572	21(17.8)	35(20.1)	0.621
**Coronary artery disease**	4(4.1)	16(8.2)	0.183	5(4.2)	15(8.6)	0.146
**Chronic kidney disease**	2(2)	4(2.1)	1	2(1.7)	4(2.3)	1
**COPD-Asthma**	12(12.2)	13(6.7)	0.11	15(12.7)	10(5.7)	0.03
**Organ involvement due to BD, n(%)**	66(67.3)	112(57.7)	0.112	73(61.9)	105(60.3)	0.794
**Organ and system involvements, n(%)**						
**Oral aphta**	96(98)	191(98.5)	1	116(98.3)	171(98.3)	1
**Genital aphta**	77(78.6)	155(79.9)	0.791	96(81.4)	136(78.2)	0.507
**Papulopustular eruption**	73(74.5)	147(75.8)	0.810	90(76.3)	130(74.7)	0.762
**Erythema nodosum**	46(46.9)	86(44.3)	0.672	53(44.9)	79(45.4)	0.935
**Arthralgia**	76(77.6)	142(73.2)	0.419	91(77.1)	127(73)	0.426
**Uveitis**	48(49)	78(40.2)	0.153	54(45.8)	72(41.4)	0.458
**Neurological involvement**	6(6.1)	13(6.7)	0.85	7(5.9)	12(6.9)	0.743
**GIS involvement**	4(4.1)	10(5.2)	0.78	4(3.4)	10(5.7)	0.415
**Cardiac involvement**	0(0)	6(3.1)	0.184	0(0)	6(3.4)	0.008
**Thrombosis**	33(33.7)	54(27.8)	0.303	39(33.1)	48(27.6)	0.316
***Arterial thrombosis***	5(5.1)	10(5.2)	0.985	6(5.1)	9(5.2)	0.973
***Venous thrombosis***	25(25.5)	43(22.2)	0.523	30(25.4)	38(21.8)	0.477
**Aneurysm**	2(2)	12(6.2)	0.152	4(3.4)	10(5.7)	0.415
**Arthritis**	36(36.7)	62(32)	0.414	38(32.2)	60(34.5)	0.686
**Sacroiliitis**	6(6.1)	20(10.3)	0.236	9(7.6)	17(9.8)	0.528
**Articular involvement** *	39(39.8)	72(37.1)	0.656	44(37.3)	67(38.5)	0.833
**Pathergy positivity, n(%)** **	36(41.1)	75(43.3)	0.761	47(44.8)	64(41.3)	0.579
**HLA-B 51 positivity, n(%)** ***	17(27)	28(22)	0.451	21(26.9)	24(21.4)	0.381
**Family history,n(%)**	28(28.6)	53(27.3)	0.821	35(29.7)	46(26.4)	0.546
**Breastfeeding duration, months, median (IQR)**	3(5)	14.5(12)		6(4)	18(12)	
**Vaginaldelivery, n(%)** ****	90(93.8)	182(93.8)	0.983	108(93.1)	164(94.3)	0.691
**Medical treatments for BD, n (%)**						
**Colchicine**	67(68.4)	153(78.9)	0.04	85(72)	135(77.6)	0.280
**Azathioprine**	23(23.5)	65(33.5)	0.07	32(27.1)	56(32.2)	0.355
**Cyclosporine**	3(3.1)	3(1.5)	0.407	2(1.7)	4(2.3)	1
**TNFa inhibitor**	8(8.2)	14(7.2)	0.772	8(6.8)	14(8)	0.687
**VDI score, median (IQR**)	0(1)	0(1)	0.835	0(1)	0(1)	0.623
**BODI score, median (IQR)**	0(1)	0(1)	0.628	0(1)	0(1)	0.450

* Articular involvement: arthritis and sacroiliitis,

** Pathergy test of 271 patients is known,

*** HLA-B51 test of 197 patients is known,

**** Type of delivery of 302 patients is known,

n: number, SD: standard deviation, IQR: interquartile range, n: number, min–max: minimum–maximum, BD Behçet’s Disease, COPD: chronic obstructive pulmonary disease, GIS: gastrointestinal system, HLA-B51: human leukocyte antigen B51, TNFa: tumor necrosis factor alpha, VDI: Vasculitis damage index, BODI: Behçet’s Syndrome Overall Damage Index

**Table 3:** Effects of various variables on breastfeeding with Behçet’s disease in univariate and multivariate logistic regression analyses.

Variables	Univariate Logistic Regression Analysis			Multivariate Logistic Regression Analysis		
	OR (Exp [ß])	95% CI	p-value	OR (Exp [ß])	95% CI	p-value
**TNFa inhibitor**	0.364	0.113–1.173	0.091	0.45	0.13–−1.562	0.209
**Sacroiliitis**	0.308	0.105–0.908	**0.033**	0.276	0.091–0.836	**0.023**
**Comorbidity**	0.359	0.155–0.835	**0.017**	0.337	0.143–0.794	**0.013**

TNFa: tumor necrosis factor alpha, OR: Odds ratio, CI: confidence intervals, TNFi: tumor necrosis factor inhibitor.

## Data Availability

The datasets used in this study are available from the corresponding author on valid request.
